# Adapted full-face snorkel masks as an alternative for COVID-19 personal protection during aerosol generating procedures in South Africa: A multi-centre, non-blinded *in-situ* simulation study

**DOI:** 10.1016/j.afjem.2021.08.002

**Published:** 2021-09-13

**Authors:** Ronel Herselman, Vidya Lalloo, Veronica Ueckermann, Daniel J. van Tonder, Edwin de Jager, Sandra Spijkerman, Wanda van der Merwe, Marizane du Pisane, Fanie Hattingh, David Stanton, Ross Hofmeyr

**Affiliations:** a Head of Department, Undergraduate and Surgical Skills Laboratories, Faculty of Health Sciences, University of Pretoria, South Africa; b Division of Emergency Medicine, Faculty of Health Sciences, University of Pretoria and Steve Biko Academic Hospital, Pretoria, South Africa; c Department Internal Medicine, University of Pretoria and Steve Biko Academic Hospital, South Africa; d Faculty Operations, Faculty of Health Sciences, University of Pretoria, South Africa; e Head of Department of Anaesthesiology, University of Pretoria and Steve Biko Academic Hospital, Pretoria, South Africa; f Undergraduate Skills Laboratory, Faculty of Health Sciences, University of Pretoria, South Africa; g Division of Infectious Diseases, Faculty of Health Sciences, University of Pretoria, South Africa; h Health Solutions Africa, Cape Town, South Africa; i Netcare Education, Faculty of Emergency and Critical Care, South Africa; j Department of Anaesthesia and Perioperative Medicine, Faculty of Health Sciences, University of Cape Town, and Groote Schuur Hospital, Cape Town, South Africa

**Keywords:** Full-face snorkel mask, N95 alternatives, Personal protective equipment (PPE), COVID-19, SARS-CoV-2

## Abstract

**Introduction:**

SARS-CoV-2 has resulted in increased worldwide demand for personal protective equipment (PPE). With pressure from ongoing epidemic and endemic episodes, we assessed an adapted snorkel mask that provides full-face protection for healthcare workers (HCWs), particularly during aerosol-generating procedures. These masks have a custom-made adaptor which allows the fitment of standard medical respiratory filters. The aim of this study was to evaluate the fit, seal and clinical usability of these masks.

**Methods:**

This multicentre, non-blinded *in-situ* simulation study recruited fifty-two HCWs to don and doff the adapted snorkel mask. Negative pressure seal checks and a qualitative fit test were performed. The HCWs completed intubation and extubation of a manikin in a university skills training laboratory, followed by a web-based questionnaire on the clinical usability of the masks.

**Results:**

Whilst fit and usability data were generally satisfactory, two of the 52 participants (3.8%) felt that the mask did not span the correct distance from the nose to the chin, and 3 of 34 participants (8.8%) who underwent qualitative testing with a Bitrex test failed. The majority of users reported no fogging, humidity or irritation. It was reportedly easy to speak while wearing the mask, although some participants perceived that they were not always understood. Twenty-one participants (40%) experienced a subjective physiological effect from wearing the mask; most commonly a sensation of shortness of breath.

**Discussion:**

A fit-tested modified full-face snorkel mask may offer benefit as a substitute for N95 respirators and face shields. It is, however, important to properly select the correct mask based on size, fit testing, quality of the three-dimensional (3D) printed parts and respiratory filter to be used. Additionally, HCWs should be trained in the use of the mask, and each mask should be used by a single HCW and not shared.

## African relevance

• South Africa has to-date diagnosed more than 1,500,000 cases of SARS-CoV-2 infection. Despite several international studies on the efficacy and safety of the full-face snorkel mask as an alternative to N95 respirators, goggles and face shields, they are not commonly available or used in high-risk settings such as emergency centres and critical care units in Africa.
• Adapted full-face snorkel masks can be used as PPE by HCWs. It is, however, important to examine the development of standardized procedures for donning, doffing and decontamination of these masks in the African context.
• Local studies on these masks can contribute to creating awareness of safe and suitable alternatives amidst global shortages of traditional personal protective equipment.

## Introduction

Since the first reported case of Coronavirus Disease 2019 (COVID-19) caused by the severe acute respiratory syndrome coronavirus-2 (SARS-COV-2) in March 2020 in South Africa [1], the country and the world has experienced the same increased demand for personal protective equipment (PPE). South Africa entered its second wave of the pandemic in December 2020. National statistics reflected 1.43 million confirmed cases and 42,550 deaths on 28 January 2021. With close to 1360 reported cases of ventilated patients on that date, it is imperative to expand strategies to protect HCWs from critically ill patients and risky procedures [2].

HCWs caring for COVID-19 patients are at high risk of being exposed to the SARS-CoV-2 virus during procedures that require protection against aerosols [3], which are frequently in emergency centres, operating theatres and critical care units. [4] Aerosols are defined as very small, lightweight particles with neutral buoyancy that can remain suspended in the air. These particles are generated from the respiratory tract during active procedures that break infectious body into small enough particles [5]. In light of diminishing PPE supplies and the inability of industrial supply chains to scale up to meet the current demand, several international studies have looked at an adapted full-face snorkel mask as an alternative to N95 respirators, goggles and face shields. 6., 7., 8., 9., 10.

Protection against aerosols requires both a filter to remove particles from inhaled air, as well as barrier protection for the eyes. This is particularly important if the HCW is close to the dispersion source, such as with endotracheal intubation or extubation. According to Tack and colleagues, several advantages can be noted when the full-face snorkel mask is compared to the standard protective measurements as recommended by the World Health Organization 9., 11.. These include limitation of direct hand contact with the face, protection against large droplets, and reusability in times of equipment shortage. Reusable masks are also cheaper than single-use respirators when in frequent use.

Initial anecdotal evidence on full-face snorkel masks suggested successful negative-pressure fit-checks (inhalation with manual occlusion of the filter port), and qualitative fit-testing, but reported on failed quantitative fit-testing [12]. The use and safety of these masks became a focal point of research in the latter part of 2020, with evidence suggesting that full-face snorkel masks are an acceptable form of PPE, provided an appropriate filter is used [10]. Several studies investigated quantitative fit-testing, filter performance, carbon dioxide (CO_2_) build-up and clinical usability. 3., 6., 8. Results regarding seal, filtration and CO_2_ build-up were comparable to that of disposable N95 respirators and data from several studies suggested that the full-face snorkel mask with an airway filter meets OHSA N95 standards in protecting healthcare workers from aerosolised particles 22., 23.. A local study on the use of the full-face snorkel mask during a bronchoscopy on children with COVID-19 reported no infection amongst the team, whereas the overall staff COVID-19 infection prevalence rate exceeded 13.5% at the time of publication [21]. Visibility has been reported as good, but speech somewhat muffled. The filters used were variable. With the increase in evidence on effectiveness of the adapted full-face snorkel mask in protecting HCWs at risk for contracting COVID-19, this study assessed the fit, seal and clinical usability of the mask during simulated clinical use.

## Methods

This multicentre, non-blinded, *in-situ* simulation study tested fit, seal, and clinical usability of full-face snorkel masks during two simulated aerosol-generating procedures, namely tracheal intubation and extubation. To standardize between the two study sites, uniform checklists for donning and doffing, [13] and intubation and extubation were used (Appendix C). Simulations were performed in skills laboratories at the Universities of Pretoria and Cape Town. Prior to the simulations, sociodemographic data of participants, including gender, age, body mass index (kg/m^2^), height and weight were collected.

After Research Ethics Committee, we recruited a convenience sample of 52 clinician volunteers (medical officers, registrars and consultants) from the departments of emergency medicine, internal medicine, anaesthesiology, family medicine, orthopaedics and critical care. We also included emergency care practitioners working for a private emergency service in South Africa. Each participant provided written informed consent. The study was performed in the midst of the first wave of infections in South Africa and as such all the participants were well versed in using typical PPE that consisted of a N95 mask, face shield/goggle, gowns, aprons and gloves. None of the participants had used a full-face snorkel mask for the purpose of PPE at the onset of the study.

**Fig. B1:**
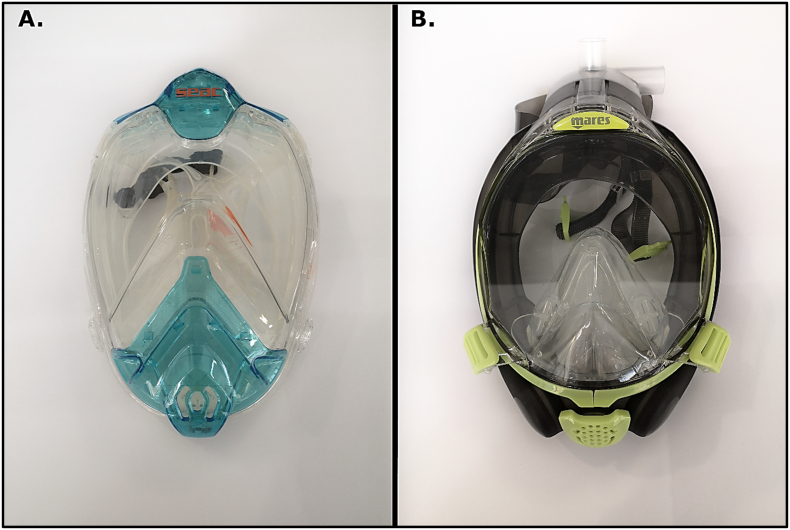
A. The SEAC Libera full face snorkel mask, and B. The Mares Sea Vu Care full face snorkel masks, used in this study.

Van Wyk, Goussard and Meintjes report on the availability of three modified full-face snorkel masks in South Africa: SEAC Libera Med+, Mares Sea Vu Care and DiveTec Paladin, with significant differences between these masks [6]. At the time of our study we had access to the SEAC Libera (SEACSUB, Italy), and the Sea Vu Care (Mares, Denmark) masks, with three available sizes of each. (See Appendix D and Fig. B1 for a comparison of the masks.)

**Fig. B2:**
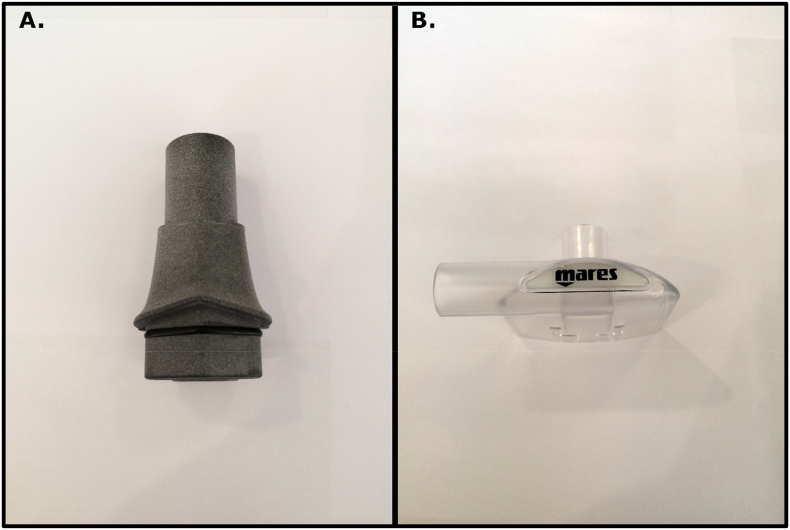
Adapter for A: SEAC Libera Med + mask and B: Mares Sea Vu Care.

Each mask was modified by removing the snorkel and fitting in a specialised adapter which allows connection of a standard respiratory filter to the inhalation and exhalation ports (Fig. B2).

**Fig. B3:**
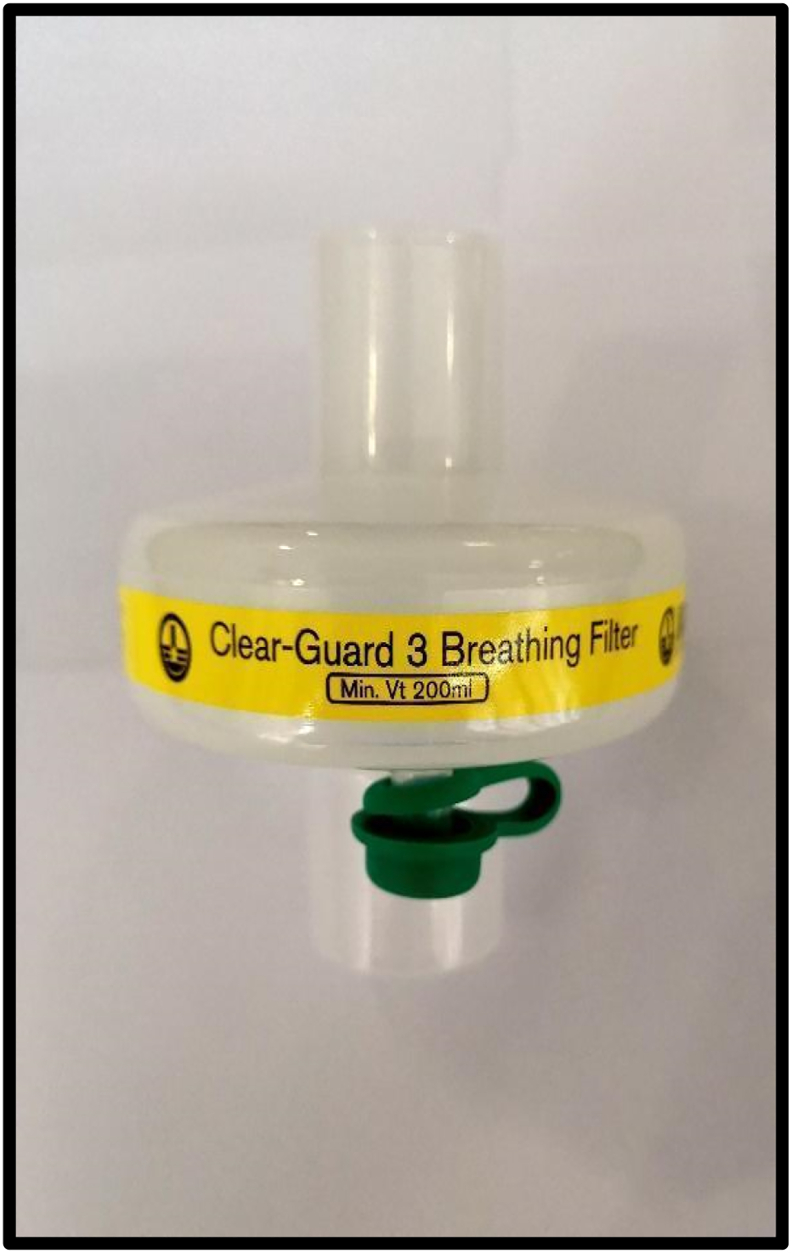
Clear-Guard 3 Breathing Filter used in this study.

The reusable adapter is 3D printed by the snorkel mask company and sold with the mask. The filter used was a hydrophobic microbial filter with >99.99% efficacy (Clear Guard, Intersurgical, South Africa) which was recommended by the mask manufacturer (Fig. B3). These filters are traditionally used in breathing circuits or exhaust valves for ventilated patients, although any respiratory filter with sufficient efficiency (such as HEPA of HMEF filters) could be substituted. That said, heat and moisture exchange is not required in this application due to the separation of inspired and expired gasses. [14] New filters were used for each participant. The masks and adapters were cleaned between participants with warm water and a mild detergent, decontaminated by full immersion in 0.1% sodium hypochlorite solution for 15 min, and air dried.

Each participant underwent fit and seal testing after a briefing on the protocol for donning, intubation, extubation and doffing. Face fit testing to determine the optimal size and strap position was conducted as per manufacturer guidelines. Participants were asked to inhale while occluding the inspiratory port with the palm of their hand. Report of a stifling sensation due to the vacuum created was considered a successful negative pressure seal test. For the qualitative fit test, participants then donned a hood from the test kit. A liquid aerosol with a bitter taste (Bitrex) was sprayed into the hood. Participants were requested to perform the following actions for 60 s each: normal breathing, deep breathing, turning their head from side to side, nodding their head up and down, reading the “Rainbow Passage” aloud, jogging on the spot, and return to normal breathing. The test was considered failed if the participant detected the bitter taste. [15]

Once the mask was considered to fit, all participants performed a simulation of a patient with COVID-19 requiring intubation. The standardized scenario included a manikin, all necessary equipment, and an assistant following accepted COVID-19 protocols. After the intubation scenario, participants were given the opportunity to perform extubation*.* Participants then completed a web-based, centrally monitored questionnaire to determine clinical usability (Appendix E). The questionnaire explored perceptions using a 5-point Likert scale [16] on the comfort of the mask, heat and humidity experienced, breathability, visual disturbances, ability to communicate with the mask on and the reusability of the mask. Tests were completed in simulation labs mimicking the advised ambient temperature of intensive care units, operating theatres or emergency centres with functioning air-conditioning systems (approximately 23 °C).

Data were summarized using descriptive statistics including frequency, mean, median, standard deviation and interquartile range.

## Results

Since the data from the Likert scale was not normally distributed, we present our results as median (IQR) values [25]. Sociodemographic characteristics of the volunteers are reported in Table 1. Half (26 of 52) were between 31 and 40 years of age, and 52% (27 of 52) were female. Thirty-five (66%) of the participants found the SEAC Libera to fit best, with 17 (33%) opting for the Mares Sea Vu Care. None of the participants reported facial hair impeding the fit of the mask. Twenty-two participants (42%) usually wear spectacles, and had to remove them when donning the mask, as the frames interfered with proper seal. However, all of these participants successfully completed intubation and extubation without their spectacles. All participants completed the study; none withdrew due to discomfort or other reasons.

**Table 1:** Baseline background and demographic characteristics of the study participants (*n* = 52).

Variables	Values
Gender	
Male	27 (51.9%)
Female	25 (48.1%)
Age in years	
20–30	14 (26.9%)
31–40	26 (50.0%)
41–50	8 (15.4%)
51–60	2 (3.8%)
60 and older	2 (3.8%)
Body mass index (kg/m^2^)	25.3 ± 5.9 (mean ± SD)

Due to high demand during the pandemic, we were unable to obtain qualitative Bitrex fit tests [17] for one study site, and thus only thirty-four participants underwent qualitative fit testing. Of these, three (8.8%) failed, with the seal becoming unstable during head movement (side-to-side or up-and-down).

The masks were well tolerated, with median values for user perception of seal and comfort of 4 and above on the 5-point Likert Scale (Table 2).

**Table 2:** Seal of the mask (*n* = 52).

Criteria	Value (median [IQR])
Acceptable size *(Likert scale: 1 (Did not fit) to 5 (Perfect fit)*	5 [1]
Adjustability of strap tension to ensure fit *(Likert scale: 1 (Could not adjust) to 5 (Excellent adjustment)*	5 [1]
Comfort over nose *(Likert scale: 1 (Not comfortable) to 5 (Comfortable)*	5 [1]
Comfort over cheeks and face *(Likert scale: 1 (Not comfortable) to 5 (Comfortable)*	5 [1]
Fit on chin *(Likert scale: 1 (Did not fit) to 5 (Proper fit)*	5 [1]
Tendency to slip *(Likert scale: 1 (Constantly slipped) to 5 (Did not slip)*	5 [0]
Ability to maintain seal when moving head*	48 (92.2%)
Proper size to span distance from nose to chin*	50 (96.2%)

Criteria that were evaluated on the 5-point Likert scale are represented as a median with interquartile range (IQR). Criteria with dichotomous (yes/no questions) * are represented as the actual number and percentage.

The average time that the mask was worn was 26.7 ± 6.5 min (mean ± SD). The self-reported scores on the clinical usability of the masks (Table 3), showed that 21 participants (40.4%) perceived a subjective physiological effect while wearing the mask. Of those 21 participants, 17 (81.0%) perceived an increase in respiratory rate, three (14.3%) described visual distortion, one each (4.8%) reported headache and chest wall fatigue. The mean time at which these physiological effects presented was 6.9 min.

**Table 3:** Clinical usability of the mask (*n* = 52).

Criteria	Value
Comfort	Median [IQR] or n (%)
Headaches*	1 (1.9%)
Ease to don the mask *Likert scale: 1 (Difficult) to 5 (Easy*)	4 [1]
Ease to doff the mask *Likert scale: 1 (Difficult) to 5 (Easy*)	5 [1]
Tension in neck after use *Likert scale: 1 (Severe tension) to 5 (No tension*)	5 [0]
Heat and humidity	Median [IQR] or n (%)
Humidity and/or drip*	50 (96.2%) No humidity 2 (1.9%) Forehead and mouth
Temperature in microenvironment*	45 (86.8%) Comfortable 7 (13.2%) Warm
Mask fogging *Likert scale: 1 (Severe fogging) to 5 (No fogging*)	5 [0]
Breathability	n (%)
Chest wall muscle fatigue*	1 (1.9%)
Increased respiratory rate*	17 (32.7%)
Increased respiratory effort*	11 (21.2%)
Visual disturbances	n (%)
Able to wear spectacles with the mask	0 (0%)
Room for eye protection adequate*	44 (84.6%)
Visual distortion	3 (5.8%)
Communication	Median [IQR]
Comfortable to talk with mask *Likert scale: 1 (Could not speak) to 5 (Spoke comfortably)*	4 [1]
Difficult to communicate with team *Likert scale: 1 (Could not communicate) to 5 (Perfect communication)*	4 [1]
Re-usability	n (%)
Visibility after repeated cleaning/decontamination*	52 (100%)
Odour/irritation after cleaning*	2 (4%)

Criteria that were evaluated on the 5-point Likert scale are represented as median and interquartile range (IQR). Criteria with dichotomous (yes/no questions)* are represented as the actual number and percentage.

Twenty-seven participants (51.9%) identified that they required assistance while donning, whilst only 10 (19.2%) participants required assistance while doffing. Using a 5-point Likert scale [16], the participants scored the ease of donning and doffing as 4 [1] and 5 [1] (median [IQR]), respectively. No cross contamination was observed during donning or doffing. None of the participants reported any fogging within the mask. Only 2 (3.8%) participants noticed humidity in the mask at the forehead and around the mouth. One participant found the temperature within the mask to be “warm”. Participants found it easy to talk with the mask while performing the simulation, scoring 4 [1] (median [IQR]) on the 5-point Likert scale. However, 16 (30.8%) participants did perceive that they were not always understood. The overall score for communication was 4 [1] (median [IQR]) on the Likert scale. After cleaning the masks, only 2 (3.8%) participants could identify an odour. No irritation due to cleaning measures was experienced by any of the participants.

## Discussion

The race to generate sustainable PPE for frontline HCWs caring for suspected or proven COVID-19 patients during high-risk procedures has overwhelmed the scientific community with a variety of novel devices purported to contain infectious aerosols 18., 19., 20.. This study evaluated fit, seal and clinical usability of adapted full-face snorkel masks as an alternative to traditional PPE. Tests were conducted in a simulated environment while performing life-saving aerosol-generating procedures.

The SEAC Libera and Mares Sea Vu Care full-face snorkel masks were used in this study (Fig. B1). We found that the main reason for requiring assistance when donning the mask was the need for help with adjustment of the silicone strap attachments. The release clips on the SEAC Libera made doffing of the mask easier. The silicone straps on both masks made for easy disinfection and quick drying.

The majority of participants (96,2%) did not find humidity to be a problem, while two participants (3.8%) reported perspiration on the forehead and mouth. We found comparable results to those previously reported for HCWs wearing spectacles, which can be attributed to factors inherent in the design which are not compatible with spectacles [9]. This did not, however, impact on the participants’ ability to complete the intubation and extubation. Fogging of masks was not a problem, correlating to the efficient airflow that is reported in similar studies [10]. Spoken communication was somewhat impaired.

Similar to the study by Tack et al., a high percentage (32.7%) of participants reported increased respiratory effort after approximately 7 min wearing the mask. Although the study performed by Tack reported slight discomfort associated with longer wearing time, the masks were generally well tolerated [9]. Our findings in terms of comfort on the nose, cheeks and chin, pressure points and securing of straps supports the notion that lightweight material improves comfort.

Although these adapted full-face snorkel masks are under assessment to be included in recommendations for PPE strategies used during aerosol-generating procedures, many HCWs have already turned to using suchlike single-piece, reusable, eye, nose and mouth protection as a useful alternative during PPE shortages [10]. With a variety of these masks already commercially available, it is important for HCWs to consider scientific evidence before using these alternatives to standard PPE. The SEAC Libera (SEACSUB, Italy) mask fitted with an Intersurgical Clear-Guard filter (Intersurgical, South Africa), meets the regulatory standard for PPE (EU regulation 425/2016). [21]. Another important consideration in terms of PPE is cost. Although we did not perform a formal cost analysis, one study on the inpatient care costs of COVID-19 in South Africa's Public Healthcare System performed a micro-costing analysis of the average inpatient costs in managing these patients. PPE (inclusive of non-sterile gloves, goggles, visors, plastic aprons, gowns, surgical face masks, and N95 respirators) was the highest driver of costs with estimates ranging between R474.50 for general wards, R711.00 for high care and R939.00 for ICU [24]. Looking at individual costs of the items, we consulted Annexure A: COVID-19 Personal Protective Equipment Price List published by the South African National Treasury on 28 April 2020. The average costs for a N95/FFP 2 mask and a visor/goggles was estimated at around R142.00. The full-face snorkel masks and adapters used in this study had a once off cost of approximately R1535.00, and a daily change of the filter at around R30.00. Although, the full-face snorkel mask is an expensive alternative to the N95 and visor we do not advocate replacement of traditional PPE, but that the full-face snorkel mask can be a viable alternative when N95s are not available. This study has several limitations. Strict COVID-19 lockdown restrictions required social distancing and indoor capacity limitations, preventing a larger number of participants from completing the study. Furthermore, the scope of this study did not include quantitative fit testing. This would more accurately reflect the true filtration efficacy of the adapted full-face snorkel mask. Several other studies have quantitatively evaluated the adapted full-face snorkel mask. 3., [5], 18. The types of filters used in these studies vary from ventilator to industrial filters. The ideal filter to be used with the adapted full-face snorkel mask is yet to be determined.

The masks were worn for the typical duration of airway management in COVID-19 patients, but not for longer periods of time required for ICU shifts or theatre cases. As such we only report on the usability of the full-face snorkel masks during airway management procedures and not clinical usability beyond the time duration in this study.

Future studies should explore the real-world utility of these masks as well as preference of participants using typical PPE *versus* the full-face snorkel mask. Furthermore, our study did not measure the level of procedural comfort for participants wearing spectacles and we suspect that wearers working shifts might not be able to use these masks. Procedural (intubation first-pass and extubation) times and success rates were not assessed in this study. However, participants mention neither any subjective prolongation of intubation time, nor unsuccessful attempts.

This study did not include objective measures of physiological discomfort. For example, a drop in oxygen saturation levels, increased inspiratory or end-tidal carbon dioxide, tachycardia or change in respiratory rate may be more objective measures of physiological impact. The study was unblinded which could result in some bias.

Future research is needed into the impact on procedural time and success rates using the snorkel mask in comparison to standard COVID-19 PPE.

Emergency centres and critical care units are generally considered high-stress environments. Intubation is often performed as a life-saving intervention. In light of the high-risk aerosol-generating nature of the procedure, novel devices that can provide a safe alternative during the COVID-19 pandemic must be investigated. Our research suggests that the adapted full-face snorkel masks were considered safe, easy to use and comfortable to wear, with limited chances of cross-contamination during donning, doffing, and when performing procedures. Finally, with the dramatic increases in scarcity and prices of N95 masks and face shields, these reusable masks are an affordable alternative.

## Dissemination of results

Results of the study will be disseminated through the academic institutions involved, and shared on various emergency medicine and critical care social media platforms so that potential users of this form of PPE may easily access the information. Feedback has been provided to each of the snorkel mask companies.

## Author contribution

Authors contributed as follow to the conception or design of the work; the acquisition, analysis, or interpretation of data for the work; and drafting the work or revising it critically for important intellectual content: RH contributed 30%; VL, VU, DJvT, and EdJ each contributed 10%; SS, WvdM, MdP, DS and FH contributed 3% and Hofmeyr 15%. All authors approved the version to be published and agree to be accountable for all aspects of the work.

## Declaration of competing interest

The authors declare no conflict of interest.

## Appendix A Supplementary data

Appendix B Fig. B1: A. The SEAC Libera full face snorkel mask, and B. The Mares Sea Vu Care full face snorkel masks, used in this study. Fig. B2: Adapter for A: SEAC Libera Med+ mask and B: Mares Sea Vu Care. Fig. B3: Clear-Guard 3 Breathing Filter used in this study. Appendix B

Appendix C Intubation and extubation checklists Appendix C

Appendix D Comparison of the different snorkel masks (adapted from van Wyk *et al*, 2020 [7])

Appendix E Google form to determine clinical usability of the full-face snorkel mask Appendix E
