# Supplementary material for: Adapted full-face snorkel masks as an alternative for COVID-19 personal protection during aerosol generating procedures in South Africa: A multi-centre, non-blinded in-situ simulation study
Source: Afr J Emerg Med. 2021 Sep 13;11(4):436–41. doi: 10.1016/j.afjem.2021.08.002 (PMC8435371; doi:10.1016/j.afjem.2021.08.002)
Supplement: Appendix B — Fig. B1: A. The SEAC Libera full face snorkel mask, and B. The Mares Sea Vu Care full face snorkel masks, used in this study. Fig. B2: Adapter for A: SEAC Libera Med+ mask and B: Mares Sea Vu Care. Fig. B3: Clear-Guard 3 Breathing Filter used in this study. [file mmc1.docx]

Appendix B:

**Figures**

Figure B1: A. The SEAC Libera full face snorkel mask, and B. The Mares Sea Vu Care full face snorkel masks, used in this study.
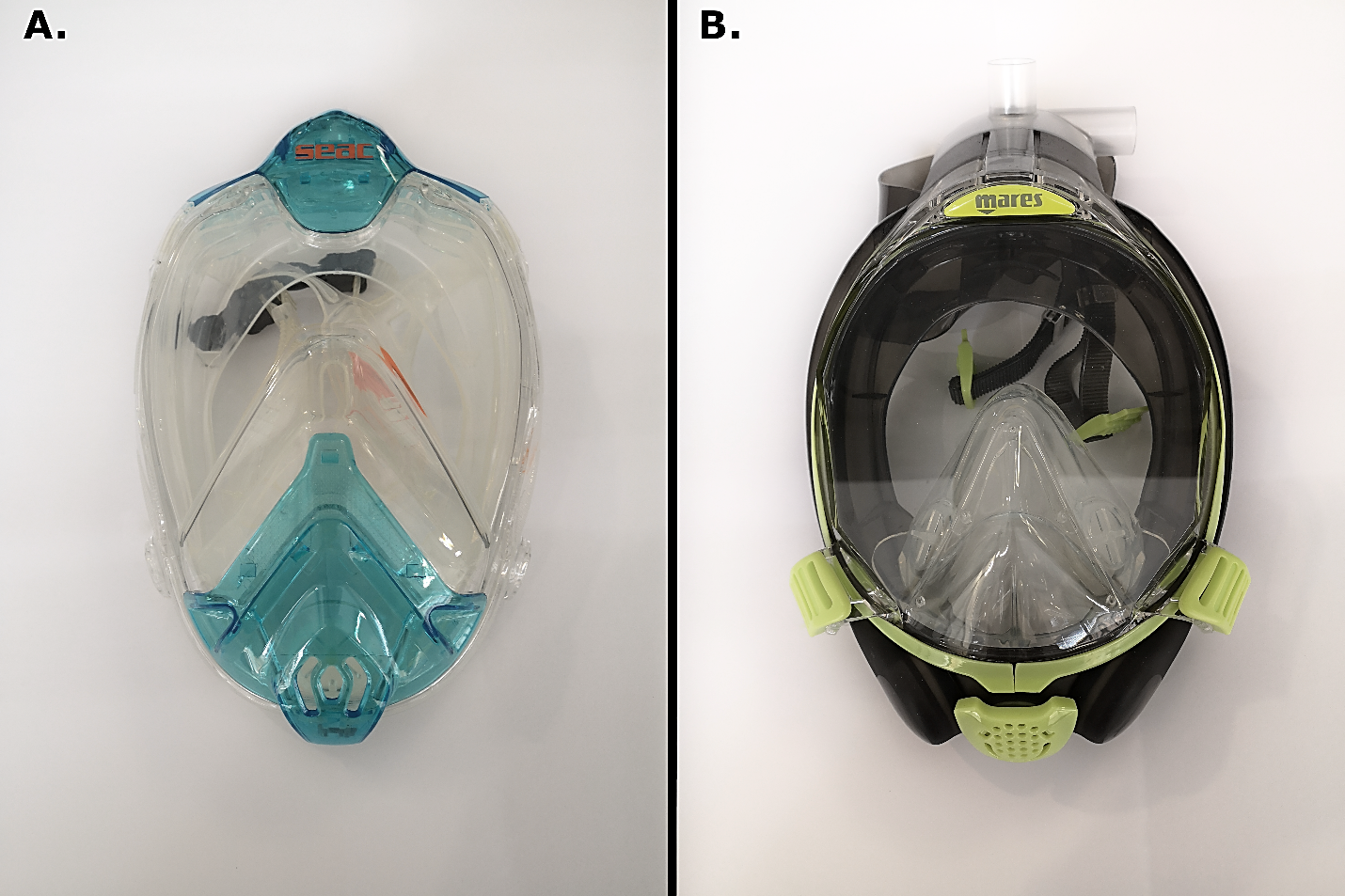


Figure B2: Adapter for **A:** SEAC Libera Med+ mask and **B:** Mares Sea Vu Care


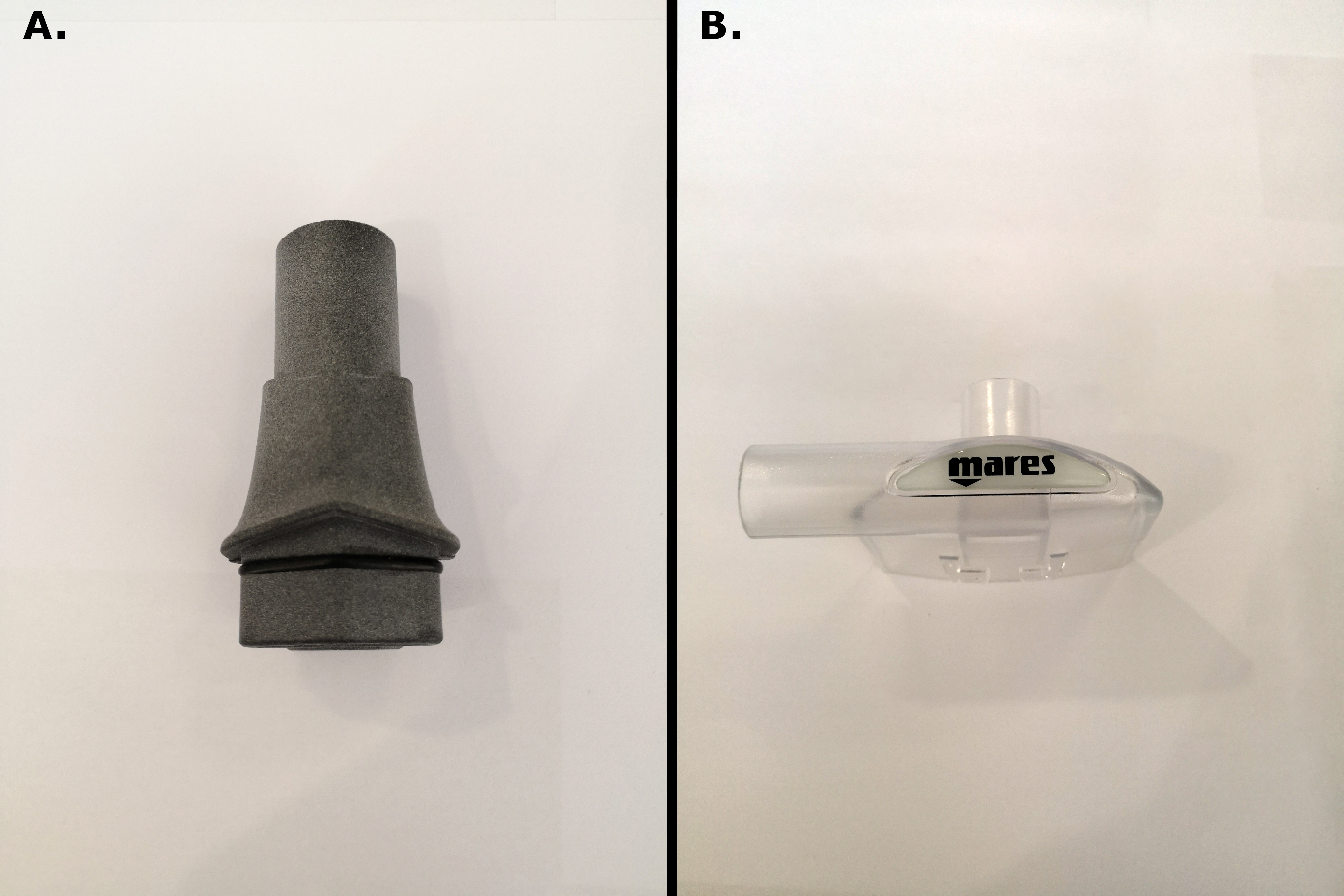


Figure B3: Clear-Guard 3 Breathing Filter used in this study


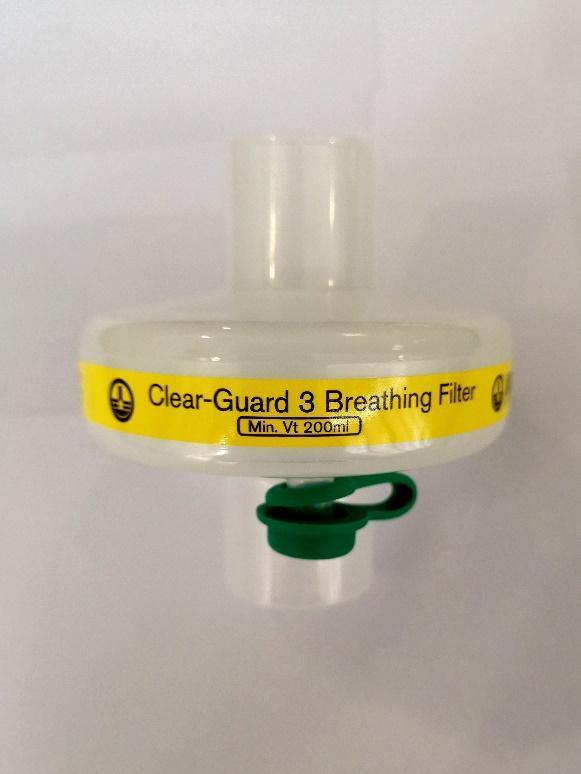


Captions:

Figure B1: **A:** SEAC Libera Med+; **B:** Mares Sea Vu Care

Figure B2: Attachment for **A:** SEAC Libera Med+ mask and **B:** Mares Sea Vu Care

Figure B3: Clear-Guard 3 Breathing Filter
