# Supplementary material for: Adapted full-face snorkel masks as an alternative for COVID-19 personal protection during aerosol generating procedures in South Africa: A multi-centre, non-blinded in-situ simulation study
Source: Afr J Emerg Med. 2021 Sep 13;11(4):436–41. doi: 10.1016/j.afjem.2021.08.002 (PMC8435371; doi:10.1016/j.afjem.2021.08.002)
Supplement: Appendix C — Intubation and extubation checklists [file mmc2.docx]

Appendix C: Intubation and extubation checklists
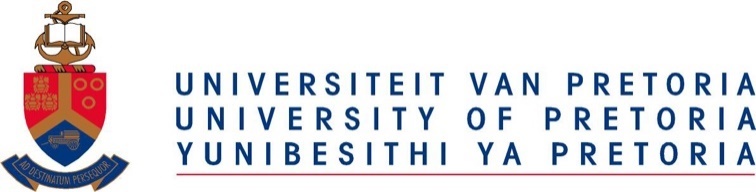


**FACULTY OF HEALTH SCIENCES**

**SKILLS LABORATORY**

### Intubation and Extubation checklist for full-face snorkel mask:

| 1 | Confirm that patient fulfils intubation criteria |
| --- | --- |
| 2 | Ventilator booked or ready (if patient is not in the same ward) |
| 3 | Internal medicine ready to take over the care of the intubated patient |
| 4 | Prepare equipment and drugs per guidelines |
| 5 | Mention special considerations for equipment and drugs ready to enter hot zone: a) back-up plan for airway - supraglottic device, two handed BVM with lowest flow and pressure, FONA, b) drugs drawn up in adequate doses and appropriately diluted where applicable, c) keep everything on a trolley covered with a linen saver |
| 6 | Assign roles for minimum airway personnel: hot zone (intubator and airway manipulator/drug administrator), outside runner and write roles on front of PPE |
| 7 | Mention special considerations for team roles: a) **intubator**: hold mask with two hands for tight seal, pass circuit to A/W manipulator when ready to intubate, intubate, inflate cuff, and strap ETT in position once placement confirmed, placement confirmed with chest rise/capnography/NO stethoscope |
| 8 | Mention special considerations for team roles: a) **A/W manipulator**: manage checklist (print and laminate, clean between patients), administer induction agent and paralytic, timer, turn flow down, receive and occlude circuit during intubation, disconnect mask and remove introducer, hold tube and connect circuit, turn flow up, connects ventilator and manipulate settings, emergency CVS support |
| 9 | Mention special considerations for team roles: a) **Runner:** spotter for donning PPE, ready with back-up drugs and equipment, recording, spotter for doffing PPE |
| 10 | Perform hand hygiene and don PPE |
| 11 | Mention special considerations for PPE: a) double gloving to minimise contamination, b) aerosol generating PPE |
|  | ***IMPLEMENTATION: Intubation*** |
| 1 | Place standard monitoring (ECG, SpO2, NIBP) |
| 2 | Remove surgical mask/oxygen mask from patient's face |
| 3 | **INTUBATOR:** Place facemask with a reservoir bag with bacterial/viral filter with two hands, ensure no leaks |
| 4 | Pre-oxygenate for 3 minutes |
| 5 | **A/W MANIPULATOR:** administer ketamine/propofol and suxamethonium/rocuronium and allow 60 seconds before intubating |
| 6 | **A/W MANIPULATOR:** close oxygen flow as soon as patient is apnoeic and remove mask when the flow is down |
| 7 | **A/W MANIPULATOR:** time 60 seconds to ensure full paralysis, do not bag-mask ventilate during this time |
| 8 | **INTUBATOR:** intubate using a videolaryngoscope if available |
| 9 | **INTUBATOR:** place blade and introducer in receiver on trolley |
| 10 | **A/W MANIPULATOR:** holds tube and connects circuit |
| 11 | **INTUBATOR:** inflates cuff |
| 12 | **A/W MANIPULATOR:** connects ventilator with inline suctioning and confirms placement with chest rise/capnography, NO auscultation |
| 13 | **INTUBATOR:** secures ETT |
| 14 | Dispose of top pair of gloves |
|  | ***IMPLEMENTATION: Extubation*** |
| 1 | Confirm that patient fulfils extubation criteria and consider antiemetics and antitussive drugs |
| 2 | All non-essential staff should exit the room |
| 3 | Hand hygiene and personal protective equipment (PPE) with aerosol generating procedure precautions are required during extubation, and for personnel entering the room for at least 30 min after extubation. |
| 4 | Oropharyngeal suctioning with vigilance as it causes aerosol generation |
| 5 | Position the patient's head at 30 degrees |
| 6 | HCW performing the extubation should be positioned behind the patient's bed to limit exposure to coughing |
| 7 | Position the ETT to one side of the mouth, closest to the HCW that will perform the extubation |
| 8 | No positive airway pressure during extubation, i.e ventilator must be switched off |
| 9 | Keep the inline suction catheter engaged whilst performing cuff deflation |
| 10 | Position a facemask with second airway filter, using a two-handed technique to ensure a seal over the mouth and nose with the ETT exiting under the facemask. |
| 11 | Deflate ETT cuff and extubate while maintaining facemask seal. |
| 12 | Discard ETT and connect circuit to the second airway filter facemask the non-rebreather valve of a self-expanding bag |
| 13 | Maintain a two-handed mask seal until regular breathing via the circuit and any immediate post-extubation coughing has subsided. |
| 14 | Staff members should confirm that PPE integrity has been maintained. |
| 15 | Doffing should only occur once the patient has been handed over to another staff member. The room requires airborne precautions for at least 30 min after an aerosol generating procedure such as extubation |
| 16 | Doff PPE and perform HH |
