# Supplementary material for: Adapted full-face snorkel masks as an alternative for COVID-19 personal protection during aerosol generating procedures in South Africa: A multi-centre, non-blinded in-situ simulation study
Source: Afr J Emerg Med. 2021 Sep 13;11(4):436–41. doi: 10.1016/j.afjem.2021.08.002 (PMC8435371; doi:10.1016/j.afjem.2021.08.002)
Supplement: Appendix D — Comparison of the different snorkel masks [file mmc3.docx]

Appendix D: Comparison of the different snorkel masks (adapted from van Wyk *et al*, 2020 [6])

| Characteristic | SEAC Libera Med+ | Mares Sea Vu Care |
| --- | --- | --- |
| **Dual inflow/outflow** | Yes | Yes |
| **Inhalation filter** | Yes | Yes |
| **Exhalation filter** | No | Yes |
| **Number of HME filters** | 1 | 2 |
| **Internal volume** | Small | Small |
| **Top strap position** | Attached to face plate | Attached to snorkel |
| **PPE certification** | Yes (Italcert) | Pending (CE) |
| **Strap material** | Silicone | Silicone |
| **Quick release buckles** | Yes | No |
| **Approximate cost of mask with adapter** | R1500 - R1650 | R1600 |

*HME=heat and moisture exchange filter; CE=Conformité Européenne*
