# Supplementary material for: Adapted full-face snorkel masks as an alternative for COVID-19 personal protection during aerosol generating procedures in South Africa: A multi-centre, non-blinded in-situ simulation study
Source: Afr J Emerg Med. 2021 Sep 13;11(4):436–41. doi: 10.1016/j.afjem.2021.08.002 (PMC8435371; doi:10.1016/j.afjem.2021.08.002)
Supplement: Appendix E — Google form to determine clinical usability of the full-face snorkel mask [file mmc4.docx]

**Appendix E:** Google form to determine clinical usability of the full-face snorkel mask

Email address *

DEMOGRAPHIC DATA

Please complete the following section.

20-30

31-40

41-50

51-60

Older than 60

Male

Female

Prefer not to say

FULL FACE SNORKEL MASK

*Required

Your email address

Date of use of mask *

Date

yyyy/mm/dd

Total time that you wore the mask (minutes) *

Your answer

Please state your age *

Please state your gender *

Yes

No

SEAC Libera

SEAC Unica

Mares Sea Vu Care

Yes

No

Please state your weight (kg) *

Your answer

Please state your height (cm) *

Your answer

Please state your body mass index (BMI) *

Your answer

Are you a smoker? *

Type of mask *

Please state the type of filter used. *

Your answer

Did you ensure that any hair growth between the skin and mask that could

interfere with the sealing surface (stubble beard, mustache, long hair, side burns)

was altered or removed? *

Intubation

Extubation

SEAL TEST

Please complete the following questions related to the seal, comfort and adequacy of the mask on a

scale from 1 to 5. (1=Bad; 5=Excellent)

Did not fit

1 2 3 4 5

Perfect fit

Could not adjust

1 2 3 4 5

Excellent adjustment

Not comfortable

1 2 3 4 5

Comfortable

Yes

No

Could not speak

1 2 3 4 5

Comfortably spoke

Please indicate the procedure you completed whilst wearing the mask *

Was the mask of an acceptable size to correctly fit your face? *

Were you able to set the strap tension to get an acceptable fit? *

Was the mask comfortable in its position on your nose? *

Was the room for eye protection adequate? *

Was it comfortable to talk with the mask? *

Not comfortable

1 2 3 4 5

Comfortable

Did not fit

1 2 3 4 5

Proper fit

Yes

No

Constantly slipped

1 2 3 4 5

Did not slip

Yes

No

Clinical usability

Please rate your experience on the clinical usability of the mask.

Mouth

Eyes

Forehead

No humidity or drip experienced.

Was the mask comfortable in its position on your cheeks and face? *

Did the mask have a proper fit on your chin? *

Was the mask of proper size to span the distance from your nose to chin? *

Did the mask have a tendency to slip? *

Were you able to maintain a seal when moving your head from side to side, and

up and down whilst wearing the mask? *

Did you experience any humidity and/or drip (please mark all that apply)? *

Chest tiredness

Chest wall muscle fatigue

Headaches

Increase in respiratory rate

Increase in respiratory effort

Visual distortion

None

Could not communicate

1 2 3 4 5

Perfect communication

Yes

No

Not applicable

Severe fog

1 2 3 4 5

No fog

Did you experience any of the following (please mark all that apply)? *

At what time did the symptoms mentioned above present?

Your answer

Please comment on the temperature in the mask/heat in the microenvironment

of the mask. *

Your answer

Was it difficult to communicate with your team? *

Were you able to wear your glasses?

Did your mask fog? *

Difficult

1 2 3 4 5

Easy

Yes

No

Difficult

1 2 3 4 5

Easy

Yes

No

Severe tension

1 2 3 4 5

No tension

1 2 3 4 5

Was it easy to don the mask? *

Did you require assistance when donning the mask? *

Was it easy to doff the mask? *

Did you require assistance when doffing the mask? *

Did you experience any tension in your neck after the use of the mask? *

Please comment on how you clean/decontaminate your mask *

Your answer

Please comment on the re-usability of the mask after several

cleaning/decontamination in terms of visibility *

Odor

Irritation

Both

None of the above

Observer section

Please review the recording of the procedure and comment on the following section

Yes

No

Send me a copy of my responses.

Never submit passwords through Google Forms.

reCAPTCHA

Privacy Terms

This form was created inside University of Pretoria. Report Abuse

Did you experience any odor or irritation following the cleaning of the mask?

Are there anything else that you would like to comment on?

Your answer

Did you notice any self-contamination by the participant during doffing? (Please

use the attached checklist for the standardized doffing procedure and include

the sheet as part of your data) *

Are there anything else that you would to comment on?

Your answer

Submit

Forms
