# Supplementary material for: Adapted full-face snorkel masks as an alternative for COVID-19 personal protection during aerosol generating procedures in South Africa: A multi-centre, non-blinded in-situ simulation study
Source: Afr J Emerg Med. 2021 Sep 13;11(4):436–41. doi: 10.1016/j.afjem.2021.08.002 (PMC8435371; doi:10.1016/j.afjem.2021.08.002)
Supplement: Appendix E — Google form to determine clinical usability of the full-face snorkel mask [file mmc5.pdf]

Full Face Snorkel Mask

*Required

Email address *

Your email address

DEMOGRAPHIC DATA

Please complete the following section.

Date of use of mask *

Date

yyyy/mm/dd

Total time that you wore the mask (minutes) *

Your answer

Please state your age *

- 20-30
- 31-40
- 41-50
- 51-60
- Older than 60

Please state your gender *

- Male
- Female
- Prefer not to say

Please state your weight (kg) *

Your answer

Please state your height (cm) *

Your answer

Please state your body mass index (BMI) *

Your answer

Are you a smoker? *

- Yes
- No

Type of mask *

- [ ] SEAC Libera
- [ ] SEAC Unica
- [ ] Mares Sea Vu Care

Please state the type of filter used. *

Your answer

Did you ensure that any hair growth between the skin and mask that could interfere with the sealing surface (stubble beard, mustache, long hair, side burns) was altered or removed? *

- Yes
- No

Please indicate the procedure you completed whilst wearing the mask *

- Intubation
- Extubation

SEAL TEST

Please complete the following questions related to the seal, comfort and adequacy of the mask on a scale from 1 to 5. (1=Bad; 5=Excellent)

Was the mask of an acceptable size to correctly fit your face? *

	1	2	3	4	5	
Did not fit	○	○	○	○	○	Perfect fit

Were you able to set the strap tension to get an acceptable fit? *

	1	2	3	4	5	
Could not adjust	○	○	○	○	○	Excellent adjustment

Was the mask comfortable in its position on your nose? *

	1	2	3	4	5	
Not comfortable	○	○	○	○	○	Comfortable

Was the room for eye protection adequate? *

- Yes
- No

Was it comfortable to talk with the mask? *

	1	2	3	4	5	
Could not speak	○	○	○	○	○	Comfortably spoke

Was the mask comfortable in its position on your cheeks and face? *

	1	2	3	4	5	
Not comfortable	○	○	○	○	○	Comfortable

Did the mask have a proper fit on your chin? *

	1	2	3	4	5	
Did not fit	○	○	○	○	○	Proper fit

Was the mask of proper size to span the distance from your nose to chin? *

- ○ Yes
- ○ No

Did the mask have a tendency to slip? *

	1	2	3	4	5	
Constantly slipped	○	○	○	○	○	Did not slip

Were you able to maintain a seal when moving your head from side to side, and up and down whilst wearing the mask? *

- ○ Yes
- ○ No

Clinical usability

Please rate your experience on the clinical usability of the mask.

Did you experience any humidity and/or drip (please mark all that apply)? *

- ☐ Mouth
- ☐ Eyes
- ☐ Forehead
- ☐ No humidity or drip experienced.

Did you experience any of the following (please mark all that apply)? *

- [ ] Chest tiredness
- [ ] Chest wall muscle fatigue
- [ ] Headaches
- [ ] Increase in respiratory rate
- [ ] Increase in respiratory effort
- [ ] Visual distortion
- [ ] None

At what time did the symptoms mentioned above present?

Your answer

Please comment on the temperature in the mask/heat in the microenvironment of the mask. *

Your answer

Was it difficult to communicate with your team? *

	1	2	3	4	5	
Could not communicate	○	○	○	○	○	Perfect communication

Were you able to wear your glasses?

- ○ Yes
- ○ No
- ○ Not applicable

Did your mask fog? *

	1	2	3	4	5	
Severe fog	○	○	○	○	○	No fog

Was it easy to don the mask? *

	1	2	3	4	5	
Difficult	○	○	○	○	○	Easy

Did you require assistance when donning the mask? *

○ Yes

○ No

Was it easy to doff the mask? *

	1	2	3	4	5	
Difficult	○	○	○	○	○	Easy

Did you require assistance when doffing the mask? *

○ Yes

○ No

Did you experience any tension in your neck after the use of the mask? *

	1	2	3	4	5	
Severe tension	○	○	○	○	○	No tension

Please comment on how you clean/decontaminate your mask *

Your answer

Please comment on the re-usability of the mask after several cleaning/decontamination in terms of visibility *

1	2	3	4	5
○	○	○	○	○

Did you experience any odor or irritation following the cleaning of the mask?

- ○ Odor
- ○ Irritation
- ○ Both
- ○ None of the above

Are there anything else that you would like to comment on?

Your answer

Observer section

Please review the recording of the procedure and comment on the following section

Did you notice any self-contamination by the participant during doffing? (Please use the attached checklist for the standardized doffing procedure and include the sheet as part of your data) *

- ☐ Yes
- ☐ No

Are there anything else that you would to comment on?

Your answer

Send me a copy of my responses.

Submit

Never submit passwords through Google Forms.

reCAPTCHA
Privacy Terms

This form was created inside University of Pretoria. Report Abuse

Google Forms
